# Smurf2 exerts neuroprotective effects on cerebral ischemic injury

**DOI:** 10.1016/j.jbc.2021.100537

**Published:** 2021-03-12

**Authors:** Haibin Liu, Shengtao Sun, Bing Liu

**Affiliations:** Department of Paediatrics, Linyi People’s Hospital, Linyi, China

**Keywords:** cerebral ischemic injury, Smurf2, YY1, HIF1α, DDIT4, neuroprotection, CCK-8, Cell Counting Kit-8, Co-IP, coimmunoprecipitation, DDIT4, DNA damage–inducible transcript 4 gene, HIF1α, hypoxia-inducible factor-1 alpha, IgG, immunoglobulin G, MCAO, middle cerebral artery occlusion, NC, negative control, oe, overexpression, OGD, oxygen–glucose deprivation, qPCR, quantitative PCR, RHOA, ras homolog gene family, member A, sh, short hairpin, Smurf2, Smad ubiquitination regulatory factor 2, TGF-β, transforming growth factor beta, TTC, triphenyltetrazolium chloride, YY1, Yin Yang 1

## Abstract

The present study aimed to explore specific mechanisms involved in mediating the neuroprotective effects of Smad ubiquitination regulatory factor 2 (Smurf2) in cerebral ischemic injury. A middle cerebral artery occlusion (MCAO) mouse model and an oxygen–glucose deprivation (OGD)–treated neuron model were developed. The expression of Smurf2, Yin Yang 1 (YY1), hypoxia-inducible factor-1 alpha (HIF1α), and DNA damage–inducible transcript 4 gene (DDIT4) was analyzed. Thereafter, the expression of Smurf2, YY1, HIF1α, and DDIT4 was altered in the MCAO mice and OGD-treated neurons. Apoptosis in tissues and cerebral infarction were assessed. In neurons, the expression of apoptosis-related proteins, viability, and apoptosis were assessed, followed by evaluation of lactate dehydrogenase leakage rate. The interaction between Smurf2 and YY1 was analyzed by coimmunoprecipitation assay and that between YY1 ubiquitination by *in vivo* ubiquitination experiment. The results showed downregulation of Smurf2 and upregulation of YY1, HIF1α, and DDIT4 in both MCAO mice and OGD-treated neurons. Smurf2 elevated YY1 ubiquitination and degradation, and YY1 increased HIF1α expression to promote DDIT4 in neurons. Overexpressed Smurf2 or downregulated YY1, HIF1α, or DDIT4 reduced the volume of cerebral infarction and apoptosis in MCAO mice, while enhancing cell viability and reducing apoptosis and lactate dehydrogenase leakage in OGD-treated neurons. In summary, our findings elucidated a neuroprotective role of Smurf2 in cerebral ischemic injury *via* inactivation of the YY1/HIF1α/DDIT4 axis.

A principle cause of fatality and morbidity on a global scale, cerebral ischemic injury is caused by hypoxic ischemic encephalopathy and acute cerebrovascular accident, which can lead to cognitive and motor impairment, neurodegenerative diseases, and even sudden death (1). After cerebral ischemic injury, disturbances in cerebral blood flow result in several biochemical and molecular processes, including calcium overload, stress signaling, neuroinflammation, oxidative and nitrative stress, leading to cellular necrosis and apoptosis (2). Furthermore, cerebral ischemic injury has been associated with neuron death (3). Neuroprotection is a key therapeutic strategy for addressing cerebral ischemic injury, but the majority of current neuroprotective agents are limited by poor efficacy or significant toxicity/side effects (4). Therefore, it is necessary to explore the molecular mechanisms underlying neuroprotection in cerebral ischemic injury and thus identify novel targets for therapeutic agent formulation.

As an evolutionary conserved and highly coordinated enzymatic cascade, protein ubiquitination plays a pivotal role in normal cell function and homeostasis maintenance (5). Smad ubiquitination regulatory factor 2 (Smurf2) is an homologous to the E6-associated protein carboxyl terminus-type E3 ubiquitin ligase, which exerts E3 ligase-dependent and E3 ligase-independent effects in numerous types of cells (6). In addition, Smurf2 is involved in various biological pathways, including cell cycle, cell cytoskeletal remodeling, and suppressing the transforming growth factor beta (TGF-β) signaling pathway (7). Yu *et al.* (8) observed that Smurf2 overexpression can promote functional recovery in rats after ischemic stroke by enhancing neuron differentiation. In another finding, it has been reported that Smurf2 plays a critical role in ubiquitination and degradation of Yin Yang 1 (YY1) in diffuse large B-cell lymphoma cells (9). YY1 serves as a dual function transcription factor, which can regulate transcriptional activation and repression of multiple genes related to various cellular processes, like cell differentiation, survival, apoptosis, autophagy, division, and DNA repair (10). Besides, it has been established in an earlier study that YY1 can induce neuronal glycolysis to trigger cerebral ischemia/reperfusion injury *in vivo* (11). Yet another previous study revealed that YY1 could stabilize hypoxia-inducible factor-1 alpha (HIF1α) to increase HIF1α expression, thus promoting tumor growth (12). In addition, a recent study has reported that HIF1α expression is increased by middle cerebral artery occlusion (MCAO) *in vivo* and oxygen–glucose deprivation (OGD) *in vitro* (13). Of note, it is documented that DNA damage–inducible transcript 4 gene (DDIT4) is a downstream target gene of HIF1α under hypoxia condition (14). DDIT4 was found to promote cardiomyocyte apoptosis and lead to ischemia-reperfusion injury (15). Considering these findings together, it was hypothesized that the Smurf2/YY1/HIF1α/DDIT4 axis modulates the development of cerebral ischemic injury, and the current study was designed to investigate this hypothesis using MCAO mouse models and OGD-treated neuron models.

## Results

### Smurf2 was downregulated in MCAO-induced mice and OGD-treated neurons

**Figure 1:**
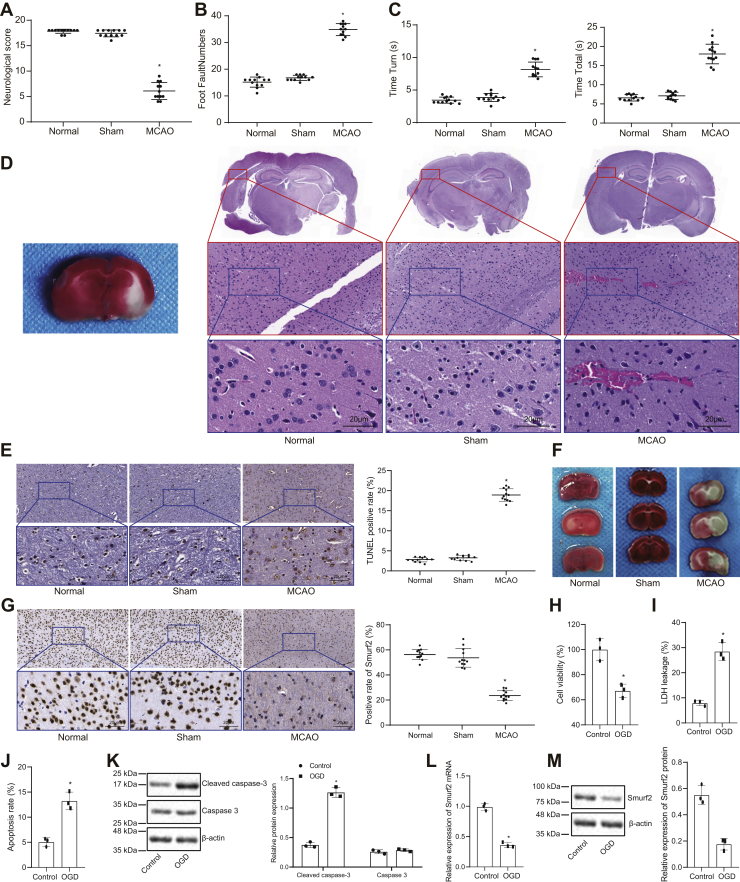
**Smurf2 downregulation is observed in MCAO-induced mice and OGD-treated neurons.***A*, neurological function score of mice after MCAO modeling. *B*, scatter plot of foot fault test of mice after MCAO modeling. *C*, scatter plot of pole test of mice after MCAO modeling. *D*, H&E staining to detect histopathological changes of ischemic penumbra (500×). *E*, TUNEL staining to detect apoptosis in ischemic penumbra of brain tissues (500×). *F*, triphenyltetrazolium chloride staining to determine cerebral infarction volume of mice. *G*, immunohistochemistry to measure Smurf2 expression in ischemic penumbra of brain tissue (500×). *H*, CCK-8 to evaluate OGD-induced neuron viability. *I*, the leakage rate of LDH in OGD-treated neurons. *J*, flow cytometry analysis of apoptosis of OGD-treated neurons. *K*, Western blot analysis of cleaved caspase 3 and caspase 3 protein expression in OGD-treated neurons. *L*, RT-quantitative PCR determination for Smurf2 mRNA expression in OGD-treated neuron model. *M*, Western blot analysis of Smurf2 protein expression in OGD-treated neuron. All measurement data were depicted as mean ± standard deviation. Data between the two groups were compared by unpaired *t* test, and comparisons between multiple groups were performed using one-way ANOVA, followed by Tukey's post hoc test. ∗*p* < 0.05 *versus* sham-operated mice or control neurons (n = 12 in the normal group, sham group, and MCAO group, and n = 3 in the control group and OGD group). CCK-8, Cell Counting Kit-8; LDH, lactate dehydrogenase; MCAO, middle cerebral artery occlusion; OGD, oxygen–glucose deprivation; Smurf2, Smad ubiquitination regulatory factor 2.

To investigate the mechanism underpinning potential neuroprotective effects of Smurf2 after cerebral ischemic injury, an MCAO mouse model and an OGD neuron model were both employed. First, to validate the successful establishment of the MCAO mouse model, neurological function scores were analyzed, which showed no significant differences between sham-operated mice and normal mice (*p* > 0.05). As compared with sham-operated mice, the neurological function score of MCAO mice was significantly reduced 24 h after operation (*p* < 0.05; Fig. 1*A*). Brain injury of mice can affect the motor function of their limbs. The results of foot fault test and pole test showed no significant difference in the number of foot faults of right limb between sham-operated mice and normal mice (*p* > 0.05). The number of foot faults of right limb was significantly higher, and T_turn_ and T_total_ were significantly longer in the MCAO mice than in sham-operated mice (*p* < 0.05; Fig. 1, *B* and *C*). It suggested that brain injury of mice can affect the motor function of their limbs. The ischemic penumbra tissues of mice in each group were selected for H&E staining, the results of which displayed no significant differences between the morphological structure of brain tissues of sham-operated mice and normal mice, and also showed that gliocyte hypertrophy and proliferation, cell and interstitial edema, and neuronal necrosis were observed in MCAO rats (Fig. 1*D*). TUNEL staining displayed that the apoptotic rate did not differ between sham-operated mice and normal mice (*p* > 0.05). The apoptotic rate of neurons in MCAO mice was significantly higher than that in sham-operated mice (*p* < 0.05; Fig. 1*E*). Based on the results of triphenyltetrazolium chloride (TTC) staining, no cerebral infarction was evident in sham-operated mice and normal mice, but the cerebral infarction volume was elevated in MCAO mice in contrast to sham-operated mice (Fig. 1*F*). These results indicated the successful establishment of MCAO mouse models. Furthermore, immunohistochemistry demonstrated that Smurf2 expression was not significantly different between sham-operated mice and normal mice (*p* > 0.05), and that MCAO mice had lower expression of Smurf2 than sham-operated mice (*p* < 0.05; Fig. 1*G*).

The OGD-treated neuron model was tested. Cell Counting Kit-8 (CCK-8) assay showed that the viability of neurons was significantly decreased after OGD treatment (*p* < 0.05; Fig. 1*H*). The results of lactate dehydrogenase (LDH) test showed an elevation of the leakage rate of LDH in OGD-treated neurons (*p* < 0.05; Fig. 1*I*). Flow cytometry demonstrated that the apoptotic rate of neurons was significantly enhanced after OGD treatment (*p* < 0.05; Fig. 1*J*). Western blot analysis presented that OGD treatment significantly elevated cleaved caspase 3 expression in neurons (*p* < 0.05), with unchanged caspase 3 expression (*p* > 0.05; Fig. 1*K*). The results of RT-quantitative PCR (RT-qPCR) and Western blot analysis depicted that the expression of Smurf2 in OGD-treated neurons was significantly reduced (*p* < 0.05; Fig. 1, *L* and *M*). Overall, Smurf2 was found to be downregulated in MCAO-induced mice and OGD-treated neurons.

### Overexpression of Smurf2 promoted neuroprotection in OGD-induced neurons

**Figure 2:**
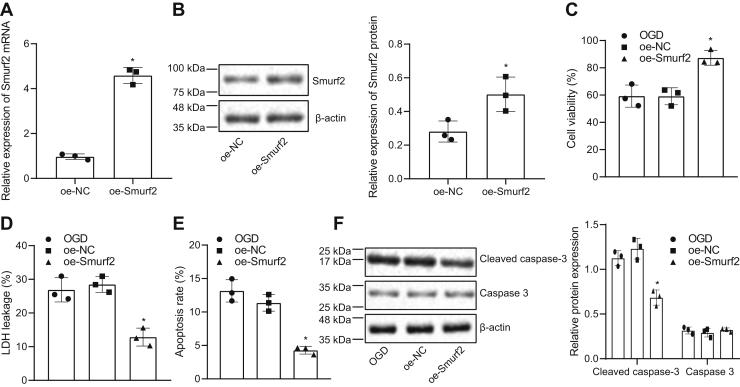
**Neuroprotection in OGD-induced neurons is promoted by overexpressing Smurf2.** OGD-treated neurons were treated or not treated with oe-NC or oe-Smurf2. *A*, the overexpression efficiency of Smurf2 in OGD-treated neurons is determined by RT-quantitative PCR. *B*, the overexpression efficiency of Smurf2 in OGD-treated neurons is measured by Western blot analysis. *C*, OGD-induced neuron viability is evaluated by Cell counting Kit-8. *D*, the leakage rate of LDH in OGD-treated neurons. *E*, flow cytometry to determine apoptosis of OGD-treated neurons. *F*, cleaved caspase 3 and caspase 3 protein expression in OGD-treated neurons assessed by Western blot analysis. All measurement data were depicted as mean ± standard deviation. Data between the two groups were compared by unpaired *t* test, and comparisons between multiple groups were performed using one-way ANOVA, followed by Tukey's post hoc test. ∗*p* < 0.05 *versus* OGD-treated neurons transfected with oe-NC. The experiment was repeated three times. LDH, lactate dehydrogenase; oe-NC, overexpression-negative control; OGD, oxygen–glucose deprivation; Smurf2, Smad ubiquitination regulatory factor 2.

Smurf2 was overexpressed in OGD-induced neurons in order to explore its specific role in cerebral ischemic injury. The efficiency of the overexpression protocol for Smurf2 was validated by RT-qPCR, Western blot analysis, and immunofluorescence. The results displayed that as compared with overexpression-negative control (oe-NC)–treated neurons, the expression of Smurf2 was significantly increased in oe-Smurf2–treated neurons (*p* < 0.05; Fig. 2, *A* and *B* and Fig. S1). As depicted in Figure 2, *C*–*E*, in OGD-treated neurons, oe-Smurf2 treatment increased cell viability but decreased the leakage rate of LDH and cell apoptosis (*p* < 0.05). Moreover, Western blot analysis manifested that Smurf2 overexpression diminished cleaved caspase 3 expression but did not change the caspase 3 expression in OGD-treated neurons (*p* < 0.05; Fig. 2*F*). These findings suggested that Smurf2 overexpression induced neuroprotection in OGD-induced neurons.

### Smurf2 promoted YY1 ubiquitination and degradation in neurons

**Figure 3:**
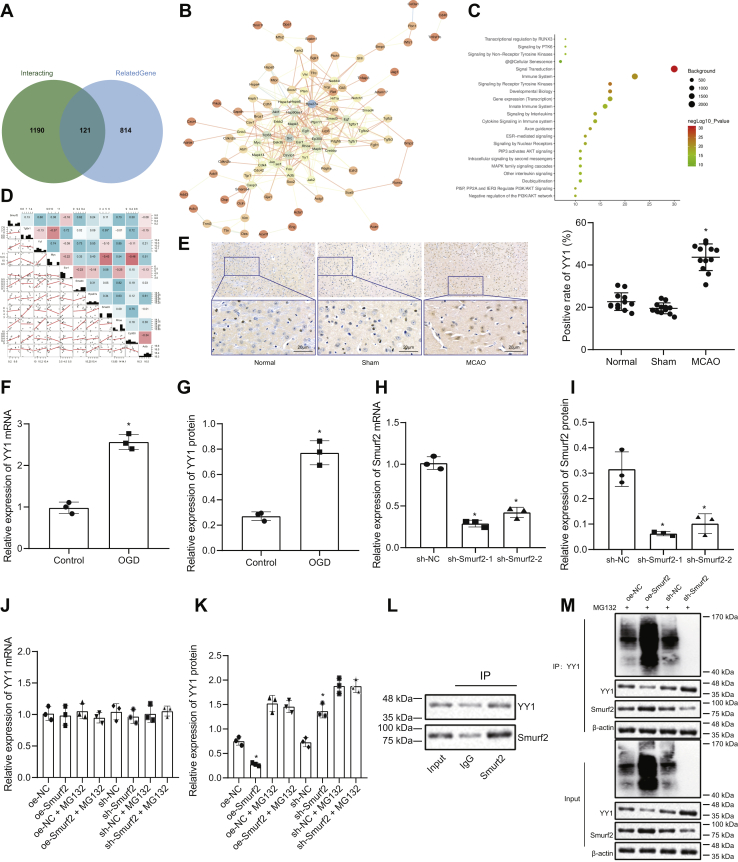
**The ubiquitination and degradation of YY1 protein is promoted by Smurf2 in neurons.***A*, Venn diagram showing intersection of the interaction genes of Smurf2 and genes related to cerebral ischemic injury in “GeneCards” database. *B*, interaction network of 121 genes using “STRING.” The *circle* color in the diagram ranges from *blue* to *orange* to represent the degreen value of genes from large to small, and the color of the lines in the figure ranges from *blue* to *orange* and from thick to thin to represent near to far coexpression relationships between genes. *C*, KOBAS-based enrichment analysis results of response pathways involving 35 genes. The *x*-axis in the figure represents the number of genes involved, and the *y*-axis represents the name of response pathway. The dot color represents −log10p value, and dot size represents the number of background genes of the response pathway. *D*, the correlation of genes in the cerebral ischemic injury–related microarray data GSE33725. The *diagonal line* in the figure represents the expression value of genes in the microarray data, the number on the *right side of the diagonal line* represents the correlation coefficient, the color ranging from *red* to *blue* represents the correlation coefficient from negative to positive, and the *left side of the diagonal line* represents the correlation map between genes. *E*, immunohistochemistry results of YY1 expression in ischemic penumbra of brain tissues of mice after MCAO modeling (500×). *F*, YY1 mRNA expression in OGD-treated neuron model detected by RT-quantitative PCR (RT-qPCR). *G*, YY1 protein expression in OGD-treated neuron model determined by Western blot analysis. *H*, Smurf2 mRNA expression in control neurons after alteration of Smurf2 determined by RT-qPCR. *I*, Western blot analysis of Smurf2 protein expression in control neurons after alteration of Smurf2. *J*, YY1 mRNA expression in neurons after alteration of Smurf2 detected by RT-qPCR. *K*, YY1 protein expression in neurons after alteration of Smurf2 determined by Western blot analysis. *L*, interaction between YY1 and Smurf2 determined by coimmunoprecipitation. *M*, YY1 ubiquitination degree in neurons after overexpression and silencing of Smurf2 determined by ubiquitination experiment. All measurement data were depicted as mean ± standard deviation. Data between the two groups were compared by unpaired *t* test, and comparisons between multiple groups were performed using one-way ANOVA, followed by Tukey's post hoc test. ∗*p* < 0.05 *versus* control neurons or neurons treated with oe-NC or sh-NC. The experiment was repeated three times. MCAO, middle cerebral artery occlusion; NC, negative control; oe, overexpression; OGD, oxygen–glucose deprivation; sh, short hairpin; Smurf2, Smad ubiquitination regulatory factor 2; YY1, Yin Yang 1.

Thereafter, the downstream mechanisms of Smurf2 were explored. The GeneCards database predicted 1311 interacting genes of E3 ubiquitin ligase Smurf2 and 935 genes associated with cerebral ischemic injury, where 121 genes were overlapping (Fig. 3*A*). Through interaction analysis of these 121 genes using “STRING,” 35 genes were found to be at the core of the network map (degree ≥ 7) (Fig. 3*B*). KOBAS software (version 3.0.3, Center for Bioinformatics, Peking University & Institute of Computing Technology, Chinese Academy of Sciences) was used to analyze the response pathways that these 35 genes participated in, which showed 11 genes were involved in the deubiquitination pathway (Fig. 3*C*). Then, 10 of these genes were also found in the expression matrix of the cerebral ischemia injury–related microarray data GSE33725. We used the expression matrix to obtain the correlation between E3 ubiquitin ligase Smurf2 and the genes (Fig. 3*D*). From Figure 3*D*, correlation coefficients between Smurf and other genes were obtained, among which YY1, SMAD4, and ras homolog gene family, member A (RHOA) had higher correlation coefficients. It was further validated in neurons that Smurf2 mediated YY1 ubiquitination and degradation. First, the expression of YY1 was determined in MCAO mice and OGD-treated neurons. Immunohistochemical results showed that there was no significant difference between the sham-operated mice and normal mice (*p* > 0.05), but as compared with sham-operated mice, YY1 expression was significantly increased in MCAO mice (*p* < 0.05; Fig. 3*E*). The results of RT-qPCR and Western blot analysis documented that YY1 expression was significantly elevated in OGD-treated neurons as compared with control neurons (*p* < 0.05; Fig. 3, *F* and *G*).

RT-qPCR and Western blot analysis were used to assess the silencing efficiency of Smurf2 in control neurons. The results showed that as compared with short hairpin (sh)-NC, the expression of Smurf2 was significantly reduced after treatment with sh-Smurf2-1 and sh-Smurf2-2 (*p* < 0.05; Fig. 3, *H* and *I*). Since the silencing efficiency of sh-Smurf2-1 was higher than that of sh-Smurf2-2, all subsequent experiments were conducted with sh-Smurf2-1 (sh-Smurf2). RT-qPCR and Western blot analysis presented that there was no significant difference in YY1 mRNA levels after different treatments (*p* > 0.05; Fig. 3*J*). YY1 protein expression was reduced by overexpressing Smurf2 and enhanced by silencing Smurf2 (*p* < 0.05), and there was no potent difference in YY1 protein expression after different treatments, when MG132, a proteasome inhibitor, was added (*p* > 0.05; Fig. 3*K*). The results of coimmunoprecipitation (Co-IP) assay exhibited that Smurf2 interacted with YY1 (Fig. 3*L*). The ubiquitination test results reported that the ubiquitination degree of YY1 increased significantly after treatment with oe-Smurf2 and diminished markedly after treatment with sh-Smurf2 (Fig. 3*M*). These results suggested that, in neurons, Smurf2 mediates the ubiquitination and degradation of YY1 protein.

### Overexpression of YY1 inhibited the neuroprotective effect of Smurf2 on OGD-treated neurons

**Figure 4:**
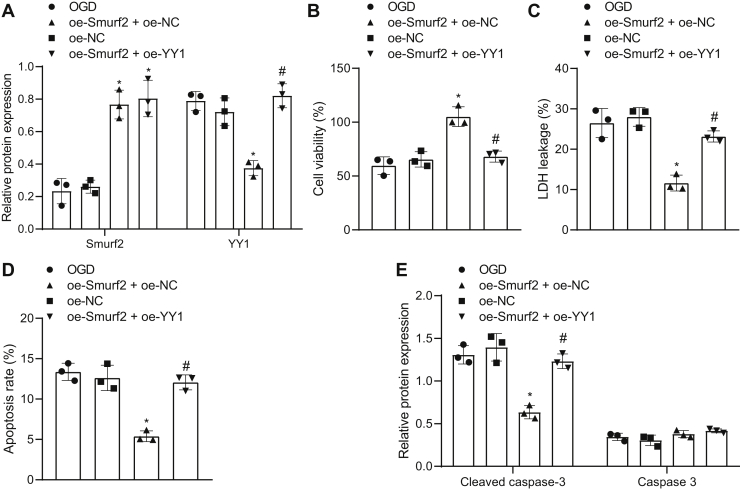
**The neuroprotective effect of Smurf2 is inhibited by YY1 on OGD-treated neurons.** OGD-treated neurons were treated or not treated with oe-NC, oe-Smurf2 + oe-NC, and oe-Smurf2 + oe-YY1. *A*, the protein expression of Smurf2 and YY1 in OGD-treated neurons is measured by Western blot analysis. *B*, OGD-induced neuron viability evaluated by Cell Counting Kit-8 assay. *C*, the leakage rate of lactate dehydrogenase in OGD-treated neurons. *D*, flow cytometry to determine apoptosis of OGD-treated neurons. *E*, cleaved caspase 3 and caspase 3 protein expression in OGD-treated neurons assessed by Western blot analysis. All measurement data were depicted as mean ± standard deviation. Comparisons between multiple groups were performed using one-way ANOVA, followed by Tukey's post hoc test. ∗*p* < 0.05 *versus* OGD-treated neurons transfected with oe-NC. #*p* < 0.05 *versus* OGD-treated neurons transfected with oe-Smurf2 + oe-NC. The experiment was repeated three times. NC, negative control; oe, overexpression; OGD, oxygen–glucose deprivation; Smurf2, Smad ubiquitination regulatory factor 2; YY1, Yin Yang 1.

We further validated the protective effect of Smurf2-mediated ubiquitination and degradation of YY1 protein on OGD-induced neurons. First, Western blot analysis showed that as compared with oe-NC treatment, the expression of Smurf2 increased significantly (*p* < 0.05) after treatment with oe-Smurf2 + oe-NC or oe-Smurf2 + oe-YY1 as compared with oe-NC treatment, whereas the expression of YY1 decreased obviously (*p* < 0.05) after treatment with oe-Smurf2 + oe-NC as compared with treatment with oe-Smurf2 + oe-NC, and the expression of YY1 was elevated significantly (*p* < 0.05) after treatment with oe-Smurf2 + oe-YY1 (Fig. 4*A*). As depicted in Figure 4, *B-D*, Smurf2 overexpression enhanced viability but reduced the leakage rate of LDH and apoptosis in OGD-treated neurons, which was abrogated by overexpression of YY1 (*p* < 0.05). The results of Western blot analysis showed that cleaved caspase 3 expression declined upon treatment with oe-Smurf2 in OGD-treated neurons, with unchanged caspase 3 expression, which was countered by additional treatment with oe-YY1 (Fig. 4*E*). Collectively, these data showed that YY1 upregulation reversed the neuroprotective effects of Smurf2 on OGD-treated neurons.

### YY1 induced neuron injury through the HIF1α/DDIT4 axis

**Figure 5:**
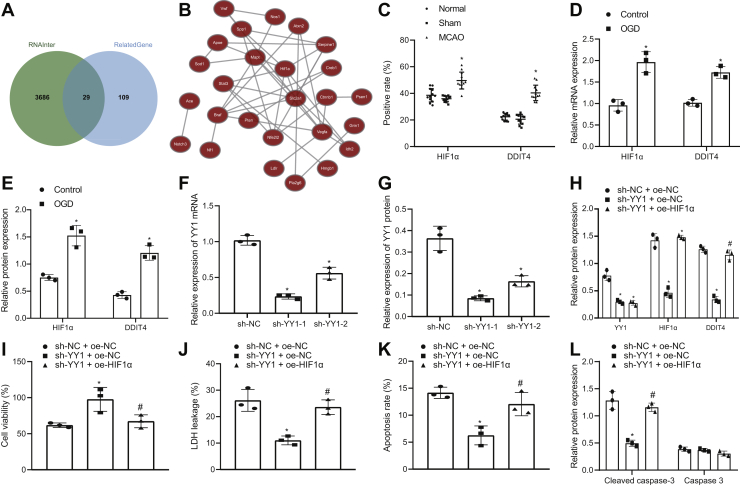
**YY1 upregulation promotes neuron injury by activating the HIF1α/DDIT4 axis.***A*, Venn diagram depicting intersection of regulatory genes of YY1 and genes related to cerebral ischemic injury in RNAInter Web site. *B*, network showing coexpression relationship of 29 genes in mice using “Coexpedia.” *C*, immunohistochemistry results of HIF1α and DDIT4 expression in ischemic penumbra of brain tissues of mice after MCAO modeling. *D*, HIF1α and DDIT4 mRNA expression in OGD-treated neuron model determined by RT-quantitative PCR. *E*, HIF1α and DDIT4 protein expression in OGD-treated neuron model determined by Western blot analysis. *F*, silencing efficacy of YY1 in neurons determined by RT-quantitative PCR. *G*, Western blot analysis of silencing efficacy of YY1 in neurons. OGD-treated neurons were transfected with sh-NC + oe-NC, sh-YY1 + oe-NC, and sh-YY1 + oe-DDIT4. *H*, YY1, HIF1α, and DDIT4 protein expression in neurons after YY1 silencing and DDIT4 overexpression detected by Western blot analysis. *I*, OGD-induced neuron viability evaluated by Cell Counting Kit-8 assay. *J*, the leakage rate of lactate dehydrogenase in OGD-treated neurons. *K*, flow cytometry to determine apoptosis of OGD-treated neurons. *L*, cleaved caspase 3 and caspase 3 protein expression in OGD-treated neurons assessed by Western blot analysis. All measurement data were depicted as mean ± standard deviation. Comparisons between multiple groups were performed using one-way ANOVA, followed by Tukey's post hoc test. ∗*p* < 0.05 *versus* control neurons or OGD-treated neurons transfected with sh-NC or sh-NC + oe-NC. #*p* < 0.05 *versus* OGD-treated neurons transfected with sh-YY1 + oe-NC. The experiment was repeated three times. DDIT4, DNA damage–inducible transcript 4 gene; HIF1α, hypoxia-inducible factor-1 alpha; MCAO, middle cerebral artery occlusion; NC, negative control; oe, overexpression; sh, short hairpin; YY1, Yin Yang 1.

To further predict the downstream mechanisms of YY1, 3741 regulatory genes of YY1 and 138 genes related to cerebral ischemic injury were identified using “RNAInter,” where 29 genes were found overlapping (Fig. 5*A*). The coexpression of 29 genes in mice was assessed using “Coexpedia” (Fig. 5*B*), where HIF1α (HIF1A) was found to score the highest (Table 1). Immunohistochemistry displayed no significant differences between sham-operated mice and normal mice (*p* > 0.05) and showed that the expression of HIF1α and DDIT4 was significantly higher in MCAO mice than in sham-operated mice (*p* < 0.05; Fig. 5*C*). RT-qPCR and Western blot analysis showed that the expression of HIF1α and DDIT4 in OGD-treated neurons was significantly higher than those in control neurons (*p* < 0.05; Fig. 5, *D* and *E*). These results indicated that HIF1α and DDIT4 were highly expressed in MCAO mice and OGD-treated neurons.

**Table 1:** Gene coexpression assessment score in Coexpedia web site

Rank	Gene symbol and name	Score
1	HIF1α	22.597
2	Slc2a1	16.539
3	Vegfa	14.6
4	Stat3	11.2
5	Nfe2l2	10.869
6	Braf	8.66
7	Serpine1	8.583
8	Spp1	7.958
9	Mapt	7.009
10	Ctnnb1	6.583
11	Idh2	6.521

To further test whether YY1 promoted HIF1α/DDIT4 expression, first, the silencing efficiency of YY1 was determined by RT-qPCR and Western blot analysis. The results showed that YY1 expression was notably diminished by treatment with sh-YY1-1 or sh-YY1-2 (*p* < 0.05; Fig. 5*F*). As the silencing efficiency of sh-YY1-1 was higher than that of sh-YY1-2, subsequent experiments were conducted with sh-YY1-1 (sh-YY1). The Western blot analysis showed that in comparison with treatment with sh-NC + oe-NC, the expression of YY1, HIF1α, and DDIT4 was significantly reduced after treatment with sh-YY1 + oe-NC (*p* < 0.05), and the expression of HIF1α and DDIT4 was significantly elevated after treatment with sh-YY1 + oe-HIF1α as compared with treatment with sh-YY1 + oe-NC (*p* < 0.05; Fig. 5*G*). These results demonstrated that YY1 promoted DDIT4 expression by promoting HIF1α.

To further verify whether YY1 induced neuron injury through the HIF1α/DDIT4 axis, first, the expression of YY1, HIF1α, and DDIT4 was evaluated by Western blot analysis. The results documented that compared with treatment with sh-NC + oe-NC, treatment with sh-YY1 + oe-NC significantly reduced the expression of YY1, HIF1α, and DDIT4 (*p* < 0.05) and, in comparison to treatment with sh-YY1 + oe-NC, the expression of DDIT4 was significantly enhanced (*p* < 0.05), whereas YY1 and HIF1α showed no significant difference after treatment with sh-YY1 + oe-DDIT4 (*p* > 0.05; Fig. 5*H*). Cell viability was found to be increased, but the leakage rate of LDH and apoptosis declined in OGD-treated neurons upon silencing of YY1, which was normalized by additional treatment with oe-DDIT4 (*p* < 0.05; Fig. 5, *I*−*K*). Western blot analysis depicted a downregulation of cleaved caspase 3 and unchanged caspase 3 expression in OGD-treated neurons following YY1 silencing, which was reversed by overexpressing DDIT4 (Fig. 5*L*). Together, these data indicated that YY1 activated the HIF1α/DDIT4 axis to promote neuron injury.

### Overexpression of DDIT4 repressed the neuroprotective effect of Smurf2 on OGD-induced neurons

**Figure 6:**
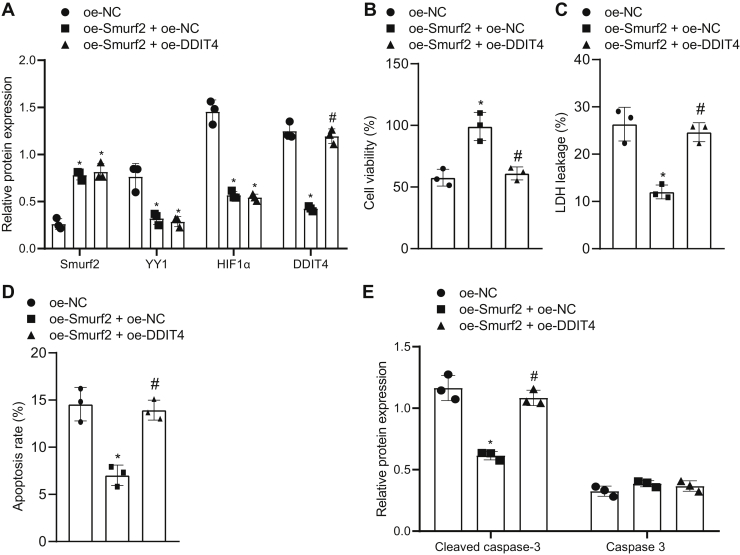
**DDIT4 upregulation reverses the neuroprotective effect of Smurf2 on OGD-induced neurons.** OGD-treated neurons were treated or not treated with oe-NC, oe-Smurf2 + oe-NC, and oe-Smurf2 + oe-DDIT4. *A*, the protein expression of Smurf2, YY1, HIF1α, and DDIT4 in OGD-treated neurons is measured by Western blot analysis. *B*, OGD-induced neuron viability evaluated by Cell Counting Kit-8 assay. *C*, the leakage rate of lactate dehydrogenase in OGD-treated neurons. *D*, flow cytometry to determine apoptosis of OGD-treated neurons. *E*, cleaved caspase 3 and caspase 3 protein expression in OGD-treated neurons assessed by Western blot analysis. All measurement data were depicted as mean ± standard deviation. Comparisons between multiple groups were performed using one-way ANOVA, followed by Tukey's post hoc test. ∗*p* < 0.05 *versus* OGD-treated neurons transfected with oe-NC. #*p* < 0.05 *versus* OGD-treated neurons transfected with oe-Smurf2 + oe-NC. The experiment was repeated three times. DDIT4, DNA damage–inducible transcript 4 gene; HIF1α, hypoxia-inducible factor-1 alpha; NC, negative control; oe, overexpression; OGD, oxygen–glucose deprivation; Smurf2, Smad ubiquitination regulatory factor 2; YY1, Yin Yang 1.

It was further verified if Smurf2 could inhibit DDIT4 expression through the YY1/HIF1α axis and exert neuroprotective effects on OGD-induced neurons. Western blot analysis documented that as compared with oe-NC treatment, the expression of Smurf2 was significantly elevated and the expression of YY1, HIF1α, and DDIT4 was significantly lowered (*p* < 0.05) upon treatment with oe-Smurf2 + oe-NC; as compared with the treatment with oe-Smurf2 + oe-NC, the expression of Smurf2, YY1, and HIF1α did not significantly differ after treatment with oe-Smurf2 + oe-DDIT4 (*p* > 0.05), whereas the expression of DDIT4 was significantly augmented (*p* < 0.05; Fig. 6*A*). The CCK-8, LDH determination, and flow cytometry assays indicated that upon overexpressing Smurf2, cell viability was enhanced, but the leakage rate of LDH and apoptosis were reduced in OGD-treated neurons, which was counteracted by additional treatment with oe-DDIT4 (*p* < 0.05; Fig. 6, *B*–*D*). Western blot analysis showed that cleaved caspase 3 expression was decreased, but caspase 3 expression did not significantly differ after overexpression of Smurf2, which was reversed by overexpression of DDIT4 (Fig. 6*E*). These findings reflected that overexpressing DDIT4 in OGD-induced neurons inhibited the neuroprotective effect of Smurf2.

### Overexpression of DDIT4 inhibits the neuroprotective effect of Smurf2 after cerebral ischemic injury in mice

**Figure 7:**
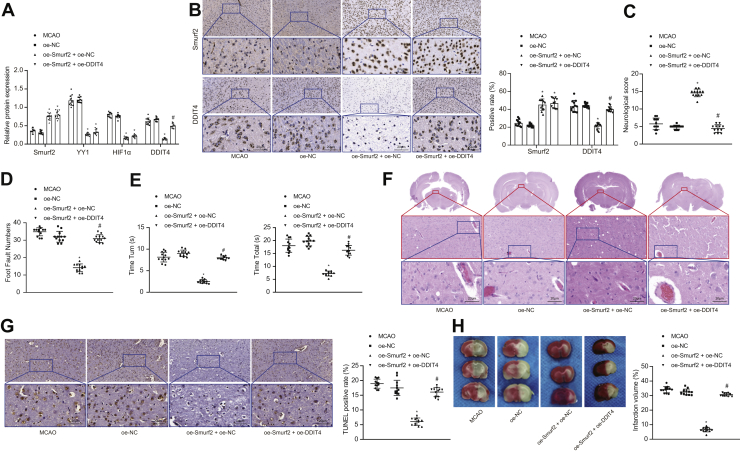
**DDIT4 upregulation reverses the neuroprotective effect of Smurf2 in mice with cerebral ischemic injury.** MCAO mice were treated or not treated with oe-NC, oe-Smurf2 + oe-NC, and oe-Smurf2 + oe-DDIT4. *A*, the protein expression of Smurf2, YY1, HIF1α, and DDIT4 in cerebral cortex of MCAO mice is measured by Western blot analysis. *B*, immunohistochemistry results of HIF1α and DDIT4 expression in ischemic penumbra of brain tissues of mice after MCAO modeling (500×). *C*, neurological function score of MCAO mice. *D*, scatter plot of foot fault test of MCAO mice. *E*, scatter plot of pole test of MCAO mice. *F*, H&E staining to detect brain histopathological changes of MCAO mice (500×). *G*, TUNEL staining to detect apoptosis in brain tissues of MCAO mice (500×). *H*, triphenyltetrazolium chloride staining to determine cerebral infarction volume of MCAO mice. All measurement data were depicted as mean ± standard deviation. Comparisons between multiple groups were performed using one-way ANOVA, followed by Tukey's post hoc test. ∗*p* < 0.05 *versus* MCAO mice treated with oe-NC. #*p* < 0.05 *versus* MCAO mice treated with oe-Smurf2 + oe-NC (n = 12). DDIT4, DNA damage–inducible transcript 4 gene; HIF1α, hypoxia-inducible factor-1 alpha; MCAO, middle cerebral artery occlusion; NC, negative control; oe, overexpression; Smurf2, Smad ubiquitination regulatory factor 2; YY1, Yin Yang 1.

Following intraventricular injection of adenovirus into mice, the expression of Smurf2, YY1, HIF1α, and DDIT4 in the cerebral cortex of MCAO model mice was evaluated using Western blot analysis. As compared with oe-NC treatment, the expression of Smurf2 was significantly higher, whereas the expression of YY1, HIF1α, and DDIT4 was lower in MCAO mice (*p* < 0.05) after treatment with oe-Smurf2 + oe-NC; as compared with treatment with oe-Smurf2 + oe-NC, treatment with oe-Smurf2 + oe-DDIT4 did not significantly influence the expression of Smurf2, YY1, and HIF1α (*p* > 0.05) but elevated the expression of DDIT4 (*p* < 0.05; Fig. 7*A*).

Immunohistochemistry showed that Smurf2 expression was elevated, but DDIT4 expression was diminished in brain tissues of MCAO mice treated with oe-Smurf2 + oe-NC. In the presence of oe-Smurf2, oe-DDIT4 did not impact Smurf2 expression and caused DDIT4 upregulation in the brain tissues of MCAO rats (Fig. 7*B*).

As displayed in Figure 7*C*, the neurological score of MCAO mice 24 h after operation was significantly enhanced upon overexpressing Smurf2, which was abolished by treatment with oe-DDIT4 (*p* < 0.05). The foot fault test and pole test showed that Smurf2 overexpression diminished the number of foot faults of right limb, T_turn_ and T_total_ in MCAO mice, which was negated by overexpressing DDIT4 (*p* < 0.05; Fig. 7, *D* and *E*). The H&E staining showed that cell and interstitial edema and neuronal necrosis in the brain tissue of mice were slowed by treatment with oe-Smurf2 + oe-NC. In the presence of oe-Smurf2, gliocyte hypertrophy and proliferation, cell and interstitial edema, and neuronal necrosis in brain tissues of mice were aggravated upon overexpressing DDIT4 (Fig. 7*F*). TUNEL staining depicted a reduced apoptotic rate of neurons in MCAO mice after upregulating Smurf2, which was normalized by additional treatment with oe-DDIT4 (*p* < 0.05; Fig. 7*G*). TTC staining exhibited that after treatment with oe-Smurf2, cerebral infarction volume was reduced in MCAO mice, which was neutralized by overexpressing DDIT4 (*p* < 0.05; Fig. 7*H*). These results demonstrated that overexpression of DDIT4 inhibits the neuroprotective effect of Smurf2 in MCAO mice.

## Discussion

The brain is the most vulnerable organ to several conditions including ischemia (16). In particular, cerebral ischemic injury contributes to a significant proportion of neuron apoptosis and death, but the underlying mechanisms are not well understood (17). Therefore, this study aimed to investigate the effects of Smurf2 on neurons after cerebral ischemic injury putatively *via* the YY1/HIF1α/DDIT4 axis. Consequently, our results showed that Smurf2 overexpression exerted neuroprotective effects in MCAO mice and OGD-treated neurons by inhibiting neuron apoptosis *via* inhibition of YY1/HIF1α/DDIT4 axis.

Initially, our data exhibited that Smurf2 showed low expression, but YY1, HIF1α, and DDIT4 were highly expressed in MCAO mice and OGD-treated neurons. As an E3 ubiquitin ligase, Smurf2 degrades TGF-β receptor I *via* ubiquitin to negatively modulate the TGF-β signaling pathway (18). The activation of the TGF-β signaling pathway in cerebral ischemia/reperfusion injury has been reported by Lou *et al.* (19). These findings are congruent with downregulation of Smurf2 in cerebral ischemic injury as noted in our study. Smurf2 induced neuron differentiation to improve functional recovery after ischemia stroke by ubiquitinating and degrading EZH2 (8). Intriguingly, it has been elucidated that Smurf2 upregulation can decrease the YY1 protein level, although not mRNA level and that Smurf2 promoted the polyubiquitination of YY1 (20). Previous experimental data have confirmed the upregulation of YY1 in MCAO mice and OGD-treated neurons (21). In the light of these two literatures, we conducted microarray analysis to verify the relationship between Smurf2 and YY1, which showed that YY1, SMAD4, and RHOA had higher correlation coefficients. Then, that Smurf2 functioned as a promoter of YY1 ubiquitination, and degradation in neurons was further validated in neurons in our research. In the present study, YY1 was found to activate the HIF1α/DDIT4 axis in neurons. Notably, YY1 repression is reported to contribute to the decrease in the accumulation and activity of HIF1α (12), whereas others have found that DDIT4 is a target gene of HIF1α (14), which further supports our results. Furthermore, HIF1α overexpression has been detected in rats with cerebral ischemic injury (22). Also, DDIT4 has been noted as upregulated in cerebral ischemic injury (23).

Experimental data from our study demonstrated that Smurf2 overexpression improved neurological function and reduced cell apoptosis in MCAO mice and also repressed apoptosis while promoting proliferation of OGD-treated neurons by inhibiting the YY1/HIF1α/DDIT4 axis. In a similar finding, Smurf2 overexpression was noted to promote neuron differentiation after a stroke (8). Another study indicated that suppression of Smurf2 led to inhibition of proliferation with promotion of apoptosis of airway smooth muscle cells in chronic asthmatic mice (24). Consistently, in a previous study, YY1 knockdown was shown to decrease ischemic brain damage and improve overall neurological functions in mice with cerebral ischemia/reperfusion injury, and YY1 overexpression was found to be involved in OGD-induced neuron apoptosis (11). Furthermore, HIF1α has been found to participate in the promoting effect of hypoxia on neuron apoptosis by reducing Bcl-2 expression (25). Sun and Yue (26) have reported that the inhibition of DDIT4 increases cell viability but decreases LDH release and cell apoptosis in neurons upon OGD/reoxygenation. Another study showed that DDIT4 silencing repressed apoptosis and LDH leakage of neurons caused by hemolysate (27). Moreover, through a series of bioinformatics analysis, we found higher correlation coefficients of Smurf2 with YY1, SMAD4, and RHOA. YY1 was chosen as the focus for this study based on reports showing YY1 can promote the occurrence of cerebral ischemic injury (21), and that Smurf2 can degrade YY1 through the ubiquitination pathway to reduce its protein expression (9). An earlier study has reported the Smad4/YY1-recognized transcriptional regulatory module in the promoter of mouse gamma-aminobutyric acid transporter subtype I gene (28). Smad4 overexpression has been identified to participate in the neuroprotective effects of exogenous activin A on OGD in PC12 cells (29). Downregulation of the RHOA/rho kinase signaling pathway was found to enhance growth-associated protein 43 and brain-derived neurotrophic factor expression to improve the inhibition of axonal regeneration and assist functional recovery after stroke, thus protecting against cerebral ischemia/reperfusion injury (30). Another report noted that bone marrow mesenchymal stem cell transplantation could improve the neurological function of rats with cerebral ischemic injury during the recovery period, possibly related to the downregulation of RHOA and rho kinase (31). Considering these reports, we speculate that the neuroprotective effect of Smurf2 might correlate to Smad4 upregulation or RHOA downregulation, which warrants the further investigation in the future.

**Figure 8:**
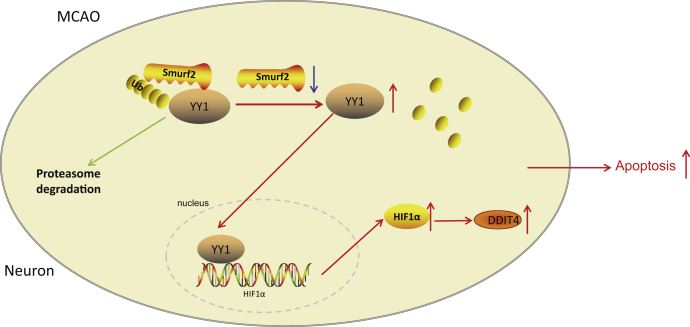
**Mechanism of Smurf2 in cerebral ischemic injury *via* YY1/HIF1α/DDIT4 axis.** Smurf2 promoted YY1 ubiquitination and degradation to decrease HIF1α and DDIT4 expression, decreasing neuron apoptosis, which exerted neuroprotective effects on cerebral ischemic injury. DDIT4, DNA damage–inducible transcript 4 gene; HIF1α, hypoxia-inducible factor-1 alpha; Smurf2, Smad ubiquitination regulatory factor 2; YY1, Yin Yang 1.

In summary, the present study demonstrated that an increase in Smurf2 inhibited the expression of YY1, HIF1α, and DDIT4. YY1 could reverse the neuroprotective effects on cerebral ischemic injury both *in vivo* and *in vitro*. Moreover, upregulated Smurf2 suppressed cerebral ischemic injury by inhibiting neuron apoptosis *via* the YY1/HIF1α/DDIT4 axis (Fig. 8), hence, providing a rational theoretical basis for clinical translation in treatments for cerebral ischemic injury. However, the underlying mechanisms mediating the interactions between Smurf2, YY1, HIF1α, and DDIT4 in cerebral ischemic injury still demand further elucidation. The strengths and limitations of the present experiment design of modeling deserve mention. The establishment of the MCAO mouse model is simple and does not require craniotomy to expose the MCA. It causes minimal injury and trauma to animals, does not affect the natural process of pathological changes in brain edema and intracranial pressure after ischemia, and avoids the randomness of emboli caused by the method of internal carotid artery injection line segment occlusion of MCA. At present, this method is considered to be the only focal cerebral ischemia model in which reperfusion injury can be observed. For MCAO modeling, there are certain surgical and technical requirements for ligating arteries and ligating wires, and large errors can occur between different operators, along with large early mortality rate of modeled animals, which need to be improved. OGD modeling is widely used as an *in vitro* model of ischemia-hypoxia disease caused by hypoxic injury of neurons, cardiomyocytes, hepatocytes, and tumor cells. OGD modeling is easy to standardize and has good repeatability and widely accepted as the best protocol for hypoxia model of cultured cells *in vitro*. It is worth noting that any cells have different morphology and performance when cultured *in vitro* compared with those *in vivo*. Therefore, the results of *in vitro* model experiments should be objectively analyzed and interpreted in combination with the results of *in vivo* experiments.

## Experimental procedures

### Ethics approval

The experimental protocols involving animals were approved by the Animal Ethics Committee of Linyi People's Hospital and were in accordance with the principles embodied in the National Institutes of Health Guide for the Care and Use of Laboratory. Efforts were made to avoid all unnecessary distress to the animals, while minimizing the number of included animals.

### MCAO model in mice

A total of 84 healthy adult male C57BL/6J mice (weighing 20–28 g, sourced from Shanghai Laboratory Animal Research Center, Shanghai, China) were maintained under standard environment conditions at 22 °C with humidity 70% and a 12 h light/12 h dark cycle. Meanwhile, the mice were fed with water and food available *ad libitum*. Among these, 60 mice were used for MCAO modeling. The MCAO mice were either treated or left untreated with adenovirus of oe-NC, oe-Smurf2 + oe-NC, or oe-Smurf2 + oe-DDIT4 (12 mice for each treatment group). The mice received intracerebroventricular injection of the corresponding adenovirus 2 h before MCAO. After anesthesia, the mice were placed prone in a stereotactic head frame (RWD Life Science). The scalp was incised along the midline, and a cavity (0.5 mm posterior and 1.0 mm lateral to the bregma) was prepared by drilling on the right side of the skull. Then, 4 μl adenovirus was injected into the ventricle (2.5 mm vertically) with a Hamilton syringe *via* a microinjection pump (KDS 310; KD Scientific Inc) at 0.2 μl/min. The needle was retained for 5 min after injection to prevent leakage. After removal of the needle, the drill hole was sealed, and the incision was sutured with stylolite. After that, the mice were allowed to recover. After 2 h, mice underwent anesthesia by intraperitoneal injection of 1% sodium pentobarbital (P3761; Sigma), and their scalps were removed. The head of the cerebral blood flow monitor was connected at the cranium of the mouse exposed ear, and a 1 cm long median longitudinal incision was made from the mandible to the sternal stalk to find the left carotid sheath, common carotid artery, external carotid artery, and internal carotid artery. Following double ligation of the proximal end of the common carotid artery, the external carotid artery was separated and ligated, and the internal carotid artery was separated. After clamping of the internal carotid artery with a microartery clamp, a small incision was made in the external carotid artery wall, the tip of the thread bolt (0.23 mm in diameter at the head and 0.18 mm in diameter at the main trunk) was inserted into the common carotid artery, and the orifice of the external carotid artery was ligated with 5/0 suture thread. After the artery clamp was removed, the thread bolt was placed in the internal carotid artery and up to the MCA (about 12.0 mm in depth), followed by covering of the wound with absorbent cotton (soaked in 0.9% sodium chloride injection) when the blood flow signal was observed with the cerebral blood flow monitor (down to about 20% as an indicator). One hour after occlusion, the suture plug was pulled out, and double ligation was applied at the entrance of the suture plug of the external carotid artery and the bifurcation of the internal carotid artery. The suture knot on the common carotid artery was united, and the blood flow was restored from the common carotid artery to the internal carotid artery. After observation of the cerebral blood flow monitor returning to 100%, the skin was sutured. In addition, 12 normal mice were utilized as normal controls, and 12 mice underwent sham operation without thread bolt in the middle artery. Subsequent to collection of the brain tissues of mice, the ischemic penumbra (Fig. S2) was selected for H&E staining, TUNEL staining, immunohistochemistry, and biochemical analysis. The temperature of mice was maintained during the operation, and the mice had free access to water and food after the operation.

### Isolation and OGD treatment of neurons

The cerebral cortex was extracted from 1-day-old newborn mice, C57BL/6J mice (sourced from Shanghai Laboratory Animal Research Center) and placed in Hank's balanced salt solution (HBSS14170112; Gibco) without Ca^2+^ and Mg^2+^ containing 1 mM sodium pyruvate (11360070; Gibco) and 10 mM 4-(2-hydroxyethyl)-1-piperazineëthanesulfonic acid (15630080; Gibco). Next, hippocampal tissues were isolated in Hank's balanced salt solution containing 0.125% trypsin (25200056; Gibco) at 37 °C for 10 min. The tissues were dispersed into single cells by repeated titration with a pasteurized tube. After digestion was terminated with DMEM (10569044; Gibco) containing 10% fetal bovine serum, the dispersed tissue was allowed to rest for 3 min. The supernatant was transferred to a new centrifuge tube at 2000 rpm for 2 min, followed by addition of the precipitate into neurobasal medium (21103049; Gibco) containing B-27 (17504044; Gibco), 0.5 mM l-glutamine (25030081; Gibco), 20 IU/ml penicillin, and 20 IU/ml streptomycin (15070063; Gibco). The cells were seeded at 4 × 10^6^ cells/well in 6-well plates coated with 100 μg/ml poly-d-lysine (A3890401; Gibco) and cultured at 37 °C with 5% CO_2_. Half of the medium was replaced with the fresh medium without glutamate every 2 to 3 days. When the purity of neurons was seen at about 95%, after 7 days of culture, the subsequent experiments were carried out.

The medium was renewed after neurons were infected with the corresponding lentivirus for 6 h. Following 18 to 24 h of culture, the neurons received OGD treatment to simulate *in vitro* neuron model with hypoxia–ischemia. After the original medium was discarded, the neurons were supplemented with glucose-free DMEM (11966025; Gibco), followed by 4-h culture in an anaerobic chamber with 95% N_2_ and 5% CO_2_. The normal control cells (control group) were cultured in DMEM (SLM-020-A; Sigma–Aldrich) for 4 h. Following OGD treatment, all neurons were normally incubated in neurobasal medium at 37 °C with 5% CO_2_ for 24 h. Then, control neurons were infected with lentivirus of oe- NC, oe-Smurf2, sh-NC, sh-Smurf2-1, or sh-Smurf2-2. Meanwhile, OGD-treated neurons were infected with lentivirus of oe-NC, oe-Smurf2 + oe-NC, oe-Smurf2 + oe-YY1, sh-NC, sh-YY1-1, sh-YY1-2, sh-NC + oe-NC, sh-YY1 + oe-NC, sh-YY1 + oe-HIF1α, sh-YY1 + oe-DDIT4, or oe-Smurf2 + oe-DDIT4. NC was considered as the lentivirus packaged with empty vector relative to the experimental group of the target gene and was used to compare the experimental effect of the target genome. All lentiviruses were bought from Shanghai Sangon Biotechnology Co, Ltd, and primer sequencing and plasmid construction were completed by Shanghai Sangon Biotechnology Co, Ltd. The experimental procedures were carried out according to the manufacturer's instructions.

### Neurological function evaluation

A neurological examination was performed 24 h after MCAO modeling by two observers, blinded to experiments. The scale consisted of six tests, including spontaneous activity, symmetry in the movement of four limbs, forepaw outstretching, climbing, body proprioception, and response to vibrissae touch. Neurological scores ranged from three scores (most severe deficit) to 18 scores (normal) (32).

### Behavioral testing

#### Pole test

At 24 h after MCAO operation, the mice were placed head upward near the top of a vertical wood pole (50 cm in length and 8 mm in diameter) with a rough surface. The test indicators included the time taken to turn the head downward completely (T_turn_) and the total time to reach the floor with all four paws (T_total_). The mice in each group were tested three times, and then the mean value was obtained.

#### Foot fault test

At 24 h after MCAO operation, the mice were placed on a horizontal grid floor above the ground and allowed to walk for 2 min. A foot fault was noted when the mouse's foot missstepped on the grid and the foot fell downward through the opening between the grids. The number of foot faults of the right limb was recorded, followed by statistical analyses.

### H&E staining

After 24 h of operation, the complete hippocampal tissue sections were selected and air dried at room temperature, followed by 30-s fixing at room temperature. After 60-s staining with hematoxylin (60 °C), the sections were differentiated with 1% alcohol hydrochloride for 3 s, and stained with eosin for 3 min, followed by 5-min dehydration with 70%, 80%, 95% ethanol, and absolute ethanol. Following 3-times immersion in xylene (5 min/times), the sections were sealed transparently with gum. A microscope (BX63; Olympus) was used to observe the extent of lesions.

### TUNEL staining

An apoptosis detection kit (C1098; Beyotime Biotechnology Co, Ltd) was employed for TUNEL staining. The sections were dewaxed in xylene for 5 min, and then fresh xylene was utilized for another 5-min dewaxing. The sections were soaked in absolute ethanol for 5 min, in 90% ethanol for 2 min, in 70% ethanol for 2 min, and in distilled water for 2 min, and reacted with 20 μg/ml protease K without DNase at 37 °C for 15 min. The 50 μl prepared TUNEL solution was added to the samples for a 60-min incubation at 37 °C in dark, followed by mounting with 4'-6-diamidino-2-phenylindole. A microscope was employed for photographing, followed by calculation of apoptosis rate. Three mice from each group were obtained, and three sections were harvested from each mouse, with five random fields chosen from each section. The cells with brown yellow nucleus were apoptotic positive cells, and the cells with blue nucleus were normal cells. The average value was taken, and the ratio of the number of brown yellow cells to that of blue cells was considered as the apoptotic rate of neurons.

### TTC staining

At 1 day after modeling, the brain tissues were obtained (the first cut was at the midpoint of the line between the anterior pole and the optic chiasm; the second cut was at the optic chiasm; the third cut was at the funnelus stalk; and the fourth cut was between the funnelus stalk and the caudal pole of the posterior lobe) followed by coronal sectioning at 2 mm interval and 20-min staining with TTC at 37 °C. After being fixed with 2% paraformaldehyde (the infarcted area was white, and the noninfarcted area was pink), brain sections were photographed with a digital camera (IXUS175; Canon). The sections were quantified using ImageJ software (version 1.46r, NIH): infarct volume = whole contralateral hemisphere volume − nonischemic ipsilateral hemisphere volume and percentage of infarct volume = infarct volume/whole contralateral hemisphere volume.

### Immunohistochemistry

Paraffin sections of mouse brain tissues were routinely dewaxed with gradient alcohol and hydrated, followed by antigen microwave thermal retrieval and blocking of endogenous peroxidase with 3% hydrogen peroxide. Then, overnight section incubation was implemented with rabbit polyclonal antibodies (Abcam) to Smurf2 (ab94483; 1:200), YY1 (ab109237; 1:100), HIF1α (ab228649; 1:100), and DDIT4 (ab106356; 1:200) at 4 °C, followed by 30-min incubation with horseradish peroxidase–labeled secondary goat anti-rabbit immunoglobulin G (IgG) antibody (ab6721: 1:10,000; Abcam) at room temperature. Subsequent to development with diaminobenzidine, hematoxylin counterstaining, and mounting, the number of positive cells was counted in five random high-power fields (×400) from each group. Positive cell rate = number of brown positive cells/total number of cells × 100%.

### Immunofluorescence

Neurons were prepared into cell slides, which were fixed with 4% paraformaldehyde for 30 min at room temperature. Cells were treated with 0.2% TritonX-100 for 15 min at room temperature and blocked with 3% bovine serum albumin for 30 min at 4 °C, followed by overnight culture with fluorescent primary rabbit anti-Smurf2 antibody (ab94483; 1:200; Abcam) in a wet box at 4 °C. Then, a 2-h cell slide culture was conducted with fluorescent goat anti-rabbit secondary antibody (ab150080; 1:500) or goat antimouse secondary antibody (ab150113; 1:200) at room temperature in the dark. The cell slides were cultured with 4'-6-diamidino-2-phenylindole (ab104139; 1:100; Abcam) in dark at room temperature for 10 min and mounted, followed by observing the results under an inverted microscope.

### RNA isolation and quantification

The total RNA in brain ischemic penumbra tissues collected 24 h after operation or neurons treated with OGD for 24 h was extracted by Trizol (16096020; Thermo Fisher Scientific, Inc), followed by reverse transcription using the First Strand cDNA Synthesis Kit (D7168L; Beyotime Biotechnology Co, Ltd). After that, RT-qPCR was performed according to the manuals of the RT-qPCR kit (Q511-02; Vazyme Biotech). PCR amplification was performed with Bio-rad real-time quantitative PCR instrument CFX96. The relative expression level of Smurf2, YY1, HIF1α, and DDIT4 was standardized by β-actin expression. These values were then exponentiated to the power of 2 (2^−ΔΔCt^) to yield fold expression relative to the reference point. The primers are depicted in Table S1.

### Western blot analysis

Radioimmunoprecipitation assay lysis reagent (R0010; Beijing Solarbio Science & Technology Co, Ltd) containing phenylmethylsulfonyl fluoride was adopted to lyse brain ischemic penumbra tissues collected 24 h after operation or neurons treated with OGD for 24 h for isolation of total protein, which was conducted according to instructions of a Nuclear and Cytoplasmic Protein Utilization Kit (P0028; Beyotime Biotechnology Co, Ltd). Following supernatant collection, a bicinchoninic acid protein assay kit (23229; Thermo Fisher Scientific) was employed for the estimation of total protein concentration, which was then adjusted into 1 μg/μl. With 100 μl samples in each tube, the samples were boiled at 100 °C for 10 min to denature the protein, which was stored at −80 °C for later use. Based on the size of the target protein band, 8% to 12% SDS gel was prepared, and the protein samples were added into each lane with the same amount with a microinjector for electrophoretic separation. The protein on the gel was electroblotted to a polyvinylidene fluoride membrane (1620177; Bio-Rad), which was sealed by 5% bovine serum albumin or 5% skim milk at room temperature for 1 h. The membrane was subsequently probed overnight at 4 °C with primary antibodies to rabbit antimouse β-actin (ab8227; 1:5000; Abcam), rabbit antimouse Smurf2 (12024; 1:1000; Cell Signaling Technology), rabbit antimouse YY1 (ab109237; 1:1000; Abcam), mouse antimouse HIF1α (ab228649; 1:1000; Abcam), rabbit antimouse DDIT4 (ab106356; 1:1000; Abcam), rabbit antimouse cleaved caspase 3 (ab49822; 1:1000; Abcam), and rabbit antimouse caspase 3 (ab13847; 1:1000; Abcam). Subsequently, the membrane was incubated with horseradish peroxidase–labeled secondary goat anti-rabbit IgG (ab6721; 1:5000; Abcam) or rabbit antimouse IgG (ab6728; 1:5000; Abcam) antibody at room temperature for 1 h. The membrane was immersed in electrogenerated chemiluminescence reaction solution (1705062; Bio-Rad) at room temperature for 1 min. After the liquid was aspirated, the membrane was covered with a preservative film, and strip exposure imaging was performed on an Image Quant LAS 4000C gel imager (GE Company). With β-actin as a loading control, the relative protein expression was expressed by the gray value of the corresponding protein bands/that of the β-actin protein band.

### CCK-8

After OGD treatment and recovery of neurons, the cell proliferation was assayed using a CCK-8 kit (GK10001; GLPBIO), according to the manufacturer's protocols. Next, 20 μl of CCK-8 was added to cells in each well, and they were incubated for 4 h. The absorbance value was measured at 450 nm, and cell proliferation curve was plotted.

### Flow cytometry

Apoptosis was detected by annexin V-FITC and propidium iodide kits (C1062L; Beyotime Biotechnology Co, Ltd). After 24 h of OGD treatment, the cells were collected in 200 μl buffer and reacted with 10 μl of annexin V-FITC and 5 μl propidium iodide for 15 min at room temperature in the dark. After supplementation with 300 μl buffer, a flow cytometer (Becton-Dickinson and Company) was used to detect apoptosis, and the apoptotic rate was calculated.

### Determination of LDH leakage rate

LDH leakage rate was determined according to the manuals of LDH cytotoxicity detection kit (CYTODET-RO; Sigma–Aldrich). The culture medium of neurons was harvested after 24-h exposure to OGD, and the supernatant was obtained by centrifugation. Cells were lysed with 1% Triton X-100 (T9284; Sigma–Aldrich), and cell fragments were removed by centrifugation to obtain a cell lysate. Cell supernatant and cell lysate were incubated with LDH reaction mixture at 37 °C for 15 min, respectively, and the reaction was stopped. Absorbance values were measured at 490 nm using a microplate reader and expressed as the percentage of released LDH to total LDH.

### Co-IP

The interaction between endogenous Smurf2 and YY1 protein was evaluated by Co-IP. The cells were lysed with Pierce IP buffer containing 1% Triton X-100, 150 mM NaCl, 1 mM ethylene diamine tetraacetic acid, 25 mM Tris–HCl (pH 7.5), followed by supplementation of protease and phosphatase inhibitors. Cell lysates were incubated overnight at 4 °C with Smurf2 antibody (sc-393848; 1:50; Santa Cruz Biotechnology) and mouse IgG antibody (3420; 1:50; Cell Signaling Technology). Thereafter, protein G beads (Dynabeads; Thermo Fisher Scientific) were added and rotated slowly at 4 °C for 8 h, followed by Western blot analysis.

### *In vivo* ubiquitination

For the determination of endogenous YY1-ubiquitination, cells were lysed in 1% SDS radioimmunoprecipitation assay buffer and treated with ultrasound. IP reaction was carried out, and mouse antimouse YY1 antibody (sc-7341; 1:50; Santa Cruz Biotechnology) were incubated with diluted cell lysate (the last 0.1% SDS) overnight at 4 °C. Protein G beads were added for 8-h incubation at 4 °C, followed by three washes in IP buffer. Ubiquitin of YY1 was detected by Western blot analysis, with rabbit antimouse ubiquitin antibody (ab7780; 1:1000; Abcam) used.

### Statistical analysis

All measurement data were depicted as mean ± standard deviation and analyzed by SPSS 21.0 software (IBM) with *p* < 0.05 as a level of statistical significance. Data from two groups were compared by unpaired *t* test, and comparisons between multiple groups were performed using one-way ANOVA, followed by Tukey's post hoc test.

## Data availability

The data sets generated and/or analyzed during the current study are available from the corresponding author on reasonable request.

## Supporting information

This article contains supporting information.

## Conflict of interest

The authors declare that they have no conflicts of interest with the contents of this article.
