# Supplementary material for: Smurf2 exerts neuroprotective effects on cerebral ischemic injury
Source: J Biol Chem. 2021 Mar 12;297(2):100537. doi: 10.1016/j.jbc.2021.100537 (PMC8363835; doi:10.1016/j.jbc.2021.100537)
Supplement: Supplemental Figures S1–S2 and Table S1 [file mmc1.pdf]

Smurf2 exerts neuroprotective effects on cerebral ischemic injury

Haibin Liu, Shengtao Sun, Bing Liu *

Department of Paediatrics, Linyi People's Hospital, Linyi 276000, China

* **Correspondence to: Bing Liu**, Department of Paediatrics, Linyi People's Hospital, No. 27, East Section of Jiefang Road, Linyi 276000, Shandong Province, China

E-mail: 13562955239@163.com

Tel.: +86-0539-8226999

Running title: Smurf2/YY1/HIF1α/DDIT4 in cerebral ischemic injury

Keywords: Cerebral ischemic injury; Smurf2; YY1; HIF1α; DDIT4; Neuroprotection

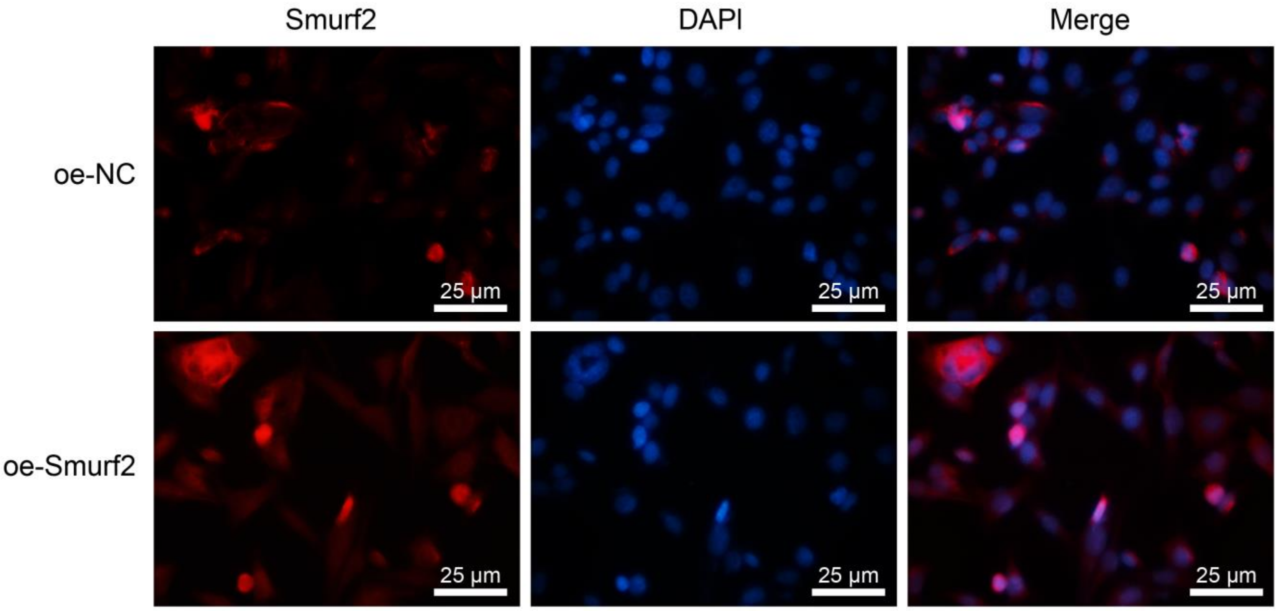

Figure S1. The overexpression efficiency of Smurf2 is confirmed by immunofluorescence (400 ×). All measurement data were depicted as mean ± standard deviation. Data between the two groups were compared by unpaired *t*-test. * $p < 0.05$ *vs.* OGD-treated neurons transfected with oe-NC. The experiment was repeated 3 times.

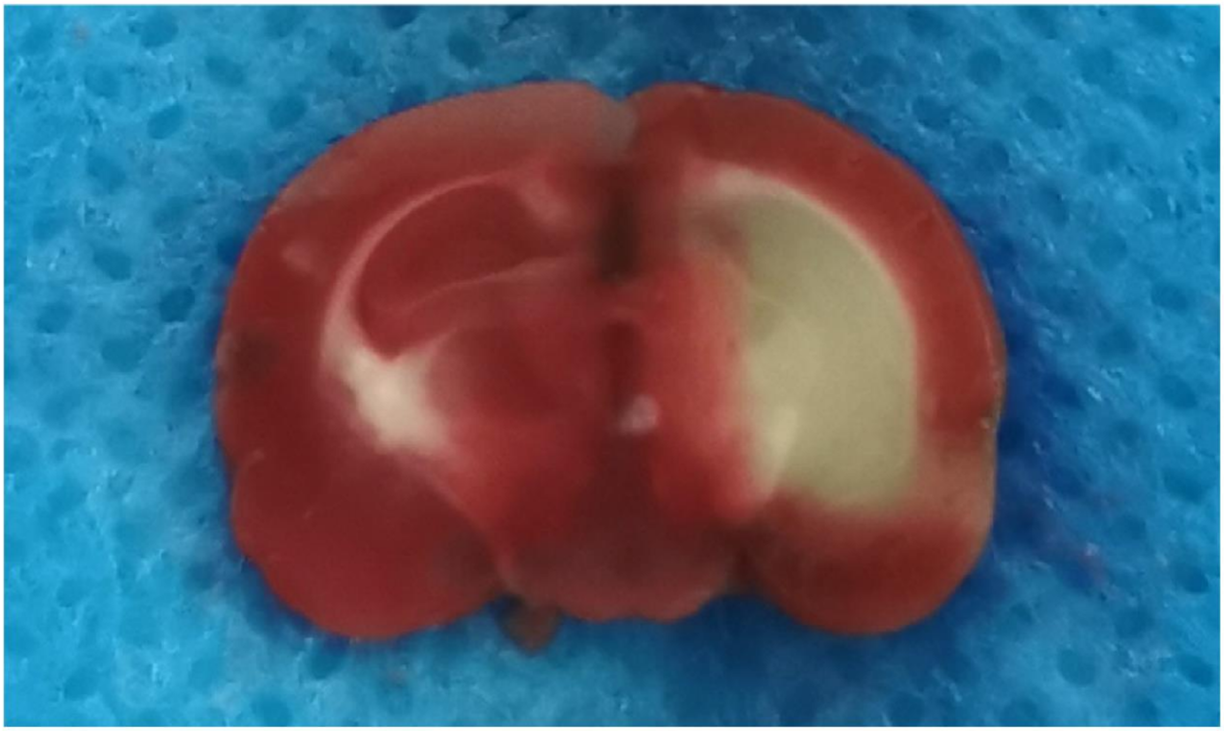

Figure S2. The anatomical location for the MCAO model establishment.

Table S1. Primer sequences for RT-qPCR

Genes	Primers (5’-3’)
Smurf2	F: CTGACAGTGCCAAGATGCAAGC<br>R: CTCATCAAATGTCTTCAGCAGG
YY1	F: TGAGAAAGCATCTGCACACC<br>R: CGCAAATTGAAGTCCAGTGA
HIF1α	F: ACCTTCATCGGAAACTCCAAAG<br>R: CTGTTAGGCTGGGAAAAGTTAGG
DDIT4	F: CAAGGCAAGAGCTGCCATAG<br>R: CCGGTACTTAGCGTCAGGG
β-actin	F: GCTCTTTTCCAGCCTTCCTT<br>R: GTGCTAGGAGCCAGAGCAGT
